# Health equity through health literacy: validating HLS_19_-Q12 in general and migrant-origin populations of Finland

**DOI:** 10.1186/s12889-026-26356-x

**Published:** 2026-01-27

**Authors:** Regina García-Velázquez, Tyler Prinkey, Eero Lilja, Robert Griebler, Thomas Link, Annamari Lundqvist, Hannamaria Kuusio, Natalia Skogberg

**Affiliations:** 1 Finnish Institute for Health and Welfare, Helsinki, Finland; 2 Austrian National Public Health Institute, Vienna, Austria

**Keywords:** Health literacy, Validity, Psychometrics, Migrant, Surveillance

## Abstract

**Background:**

Health Literacy (HL) has become popular in research for its potential in reducing health inequalities. HL is shaped by personal and situational factors, both having particular implications for migrant populations. This study adapted and validated the HLS19-Q12 in migrant and general populations of Finland using two large-scale population studies. We investigated methodological choices to ensure quality of HL measurement and monitoring across population groups. Finally, we examined the association between HL and socioeconomic, health- and migration-related factors in the general and migrant populations of Finland.

**Methods:**

We implemented the Nominal Categories Model (NCM) to empirically evaluate modeling assumptions, studied response functioning and measurement equivalence according to nativity in several language groups, including Finnish and English native (n=3673, n=379) and non-native (n=2077, n=1402) speakers, Russian native speakers (n=926). Methodological effects of opt-out and response format were investigated in the three language versions. Finally, we examined concurrent validity according to socioeconomic (i.e. sex, age, educational attainment, economic activity, economic deprivation, migrant-origin), health- (i.e. self-rated health, chronic disease, health-care use, smoking, obesity, cardiovascular disease risk score) and migration-related factors (i.e. length of residence, language proficiency, language barrier in healthcare) in the general and migrant populations under examination.

**Results:**

Cronbach’s alpha ranged from .93 to .96 and the u index indicated unidimensionality. Test information functions indicated the scale was most informative at low and medium levels of the trait continuum. The NCM showed acceptable fit and revealed expected ordering of Likert response categories, while the opt-out category showed inconsistencies. We found evidence of unbiased functioning of HLS19-Q12 respecting English and Finnish language nativity, and no evidence of acquiescence effects. Associations between HL and criterion factors were in line with expectations.

**Conclusions:**

This study introduced the Finnish version of HLS19-Q12 and approached valid and reliable HL monitoring as a key element within the field of social determinants of health. This study provided novel insights on HL while replicating findings reported in the literature. We provide specific recommendations for valid monitoring of HL and ponder over culturally and linguistically responsive policy making, in accordance with the Health in All Polices approach.

**Supplementary Information:**

The online version contains supplementary material available at 10.1186/s12889-026-26356-x.

## Background

Over the past two decades, health literacy (HL) has become a key focus in public health due to its role in preventing and managing non-communicable diseases [1]. HL is commonly defined as the ability to find, understand, evaluate, and use health information to make informed decisions about care, prevention, and health promotion. It is shaped by both personal factors—such as motivation and cognitive skills—and situational ones, including how accessible and user-friendly health services are [2–4]. While HL is often discussed in relation to individual characteristics like education or ethnicity, its connection to contextual factors such as access to information is less frequently explored [5–7]. Importantly, HL is considered more amenable than other social determinants, offering potential to reduce health inequalities [6].

For migrant populations, the relational aspect of HL has significant implications. According to the integrated conceptual framework for HL development [1], migrants are at higher risk of social and economic disadvantage and hence at a heightened risk of vulnerability. They are more likely than other population groups to encounter access barriers, such as language difficulties, limited health coverage, discrimination, and stigma. Cultural norms and religious beliefs may influence health perceptions and behaviors, while experiences before or during migration can intensify immediate health needs and widen disparities.

Language and communication barriers may delay access to healthcare services and interfere with development of therapeutic relationships with healthcare providers [8, 9]. Navigating a new healthcare system requires time and is influenced by personal factors—such as length of stay, language proficiency, and education—and the responsiveness of local services, including cultural competence and multilingual support. Limited familiarity with the system restricts access to preventive care and contributes to poorer long-term outcomes [10–12]. A European review found that migrants and refugees frequently face legal barriers, rely more often on emergency services, and have unmet needs in primary, dental and mental healthcare [13]. HL seems to have a role on how migrants engage with healthcare services and, consequently, on their health outcomes. Migrants tend to exhibit lower HL levels than local populations [14–16]. To address this gap, researchers emphasize the importance of linguistically and culturally adapted interventions, materials and personnel training [17–19]. Ensuring equitable access to understandable and actionable health information is therefore essential for advancing health equity.

Several international bodies advocate for HL monitoring as a strategy for health promotion and evaluation of public health interventions. The WHO Action Network on Measuring Population and Organizational Health Literacy [20], established in 2018, aims to strengthen HL across the WHO European Region by producing high-quality, comparable data to guide policy and practice. As part of this effort, the consortium developed instruments including the HLS_19_-Q12—a short, psychometrically sound questionnaire [21], based on the HLS-EU conceptual framework [4, 21] that has been validated in around twenty countries [21–24]. However, its use and validation in migrant populations remain limited [25].

In Finland, research has focused mainly on school-aged [26, 27] and older adults [28, 29], leaving a gap in knowledge among working-aged general and migrant populations. Valid measurement is particularly important in culturally diverse contexts, where language proficiency, response styles, or social desirability may influence survey responses, possibly biasing findings and by extension evidence-informed policymaking. In Finland, approximately 477,000 of the residents were foreign-born in 2022 at the time of data collection (9% of the total population) [30]. This figure includes individuals born outside Finland whose parents or only known parent were born abroad. In 2024, approximately 624,000 residents were born abroad or spoke a mother tongue other than the national languages (Finnish and Swedish) [30, 31]. Adults with foreign background living in Finland report significantly lower healthcare services use as compared with the general population, despite needing them to similar extent. Additionally, the foreign-background population reports higher rates of unmet medical needs [32, 33]. From the perspective of equity, efforts to ensure valid measurement of HL in this large part of the population are fundamental, particularly given the challenges they face in accessing and receiving needed healthcare.

This study validates the HLS_19_-Q12 for use in Finland, providing a brief and reliable tool suitable for national health monitoring. Both internal and external sources of validity were investigated. We draw on two large-scale population studies: the Finnish adult population’s well-being and health 2022 (referred to as Healthy Finland) [34] and the Finnish national survey on health, well-being and service use among foreign-born population 2022 (referred to as MoniSuomi) [32]. The findings seek to inform best practices for HL measurement and guide the development of culturally and linguistically responsive policymaking, in accordance with the Health in All Polices approach.

### Aims of the study

The specific aims of this study are the following:

1. To investigate psychometric functioning of the Finnish version of HLS_19_-Q12 in working-aged general and migrant populations of Finland.
2. To examine functioning of the opt-out response category across items and samples.
3. To examine response style bias in HLS_19_-Q12.
4. To assess alignment of HL scores with theoretically founded socioeconomic, health- and migration-related factors (criterion-related concurrent validity).

## Methods

### Psychometric considerations

The intended use of HLS_19_-Q12 is primarily practical: to map HL across population groups and to discriminate between groups at risk of poorer health outcomes [21]. The HLS_19_-Q12 was administered in Finland as part of large-scale population surveys conducted by the Finnish Institute for Health and Welfare. The surveys include numerous measures to assess health and well-being and thus represent a typical context for HL monitoring. We examined several psychometric properties of the questionnaire in an archetypal setting and prioritized pragmatic, applied psychometric perspectives. For a more comprehensive view on the methodological rationale of this study, we refer the reader to *Supplementary Methods 1.*

### Phase I: adaptation and translation process of the Finnish HLS_19_-Q12

In the National survey on health, well-being and service use among foreign-born population (MoniSuomi) study, the survey including the HLS_19_-Q12 was translated into 20 languages—including Finnish, English, Arabic, Russian, Estonian, and Swedish—to reflect the linguistic diversity of Finland’s migrant population. Both invitation letter and questionnaire were translated using a professional service provider (forward translation). The translated materials were reviewed by multilingual field personnel and interviewers, some of which had experience in previous population studies conducted by the Finnish Institute for Health and Welfare. Modifications were made to the translated versions according to their feedback in various iterative rounds. The adaptation process prioritized conceptual equivalence and inclusivity, targeting valid cross-cultural measurement of health literacy. The survey form including the HLS_19_-Q12 was administered both online and via paper format to ensure accessibility. The survey had a shorter version to respond via telephone interview with the multilingual field personnel, but this version did not include the HLS_19_-Q12. The Healthy Finland 2022 survey, on its behalf, was available in the two national languages, Finnish and Swedish, English and Russian.

### Phase II: data collection

The MoniSuomi survey was collected between September 2022 and March 2023 using an electronic form, which was supplemented by a paper questionnaire. The study’s sample (*n* = 18,600) was drawn using a stratified random sample from the Population Information System maintained by the Digital and Population Data Services Agency. Sampling was restricted to persons who were born abroad and were of foreign origin, who were aged 20 to 74 and had lived in Finland for at least 12 months at the time of sampling. Including all response methods, the response rate was 44.1 per cent (*n* = 7,838). Data from the Healthy Finland 2023 survey was used as comparison data for the MoniSuomi study, and both population studies were planned to be fully comparable to each other.

The Healthy Finland 2022 survey included multiple sections with varying sample selection procedures [34]. This study used data from the health examination section, which included survey, interview and laboratory data collections. The health examination sample was drawn with size *n* = 9,973. The analytic sample of this study was *n* = 3,853, which had data available for the variables we used. The subsamples of languages other than Finnish had to be combined into one composite corresponding to the foreign background participants, since their sizes were too small to be analyzed separately. Concerning MoniSuomi participants, this study includes participants who responded to the Finnish, English and Russian versions of the survey, with sample sizes showed in Table 1.

**Table 1:** Use of subsamples in this study

	Item distributions, reliability, unidimensional IRT^a^ models	MI^b^ study: equivalence of native and non-native speakers	DIF^C^ study: Finnish-origin and migrant population	DIF^C^ study: Response scale direction (two sub-studies)	Criterion validity study
Healthy Finland study: subsamples					
Finnish origin, *n* = 3673	X				X
Foreign origin, total *n* = 180: Answering to the following language versions: Finnish, *n* = 39 English, *n* = 93 Russian, *n* = 39 Swedish, *n* = 9	X		X	X1 (English) X2 (Finnish)	X
MoniSuomi study: subsamples					
Foreign origin, non-native speakers Finnish, *n* = 2077	X			X2	X
Foreign origin, native speakers English, *n* = 379	X	X		X1	X
Foreign origin, non-native speakers English, *n* = 1402	X	X		X1	X
Foreign origin, native speakers Russian, *n* = 926	X				X

^a^IRT: Item Response Theory

^b^MI: Measurement Invariance

^c^DIF: Differential Item Functioning

### Phase III: study of item distributions and item response modeling

The groups under investigation in this study are classified depending on whether they were Finnish-born speaking Finnish as their mother tongue (also referred to as Finnish origin), or foreign-born persons whose mother tongue was other than the national languages (also referred to as foreign origin and migrant). Within this group and for reasons of sample size and comparability of the two studies, we examined data from persons answering to the Russian version of the test whose mother tongue was Russian, from persons to the English version of the test whose mother tongue was English, and from persons answering to the Finnish and English versions of the questionnaire and whose mother tongue was other than these languages in which they answered. Studying the latest two groups allowed us to inspect the functioning of the test in speakers taking a test in languages other than their first (i.e. non-native speakers), which is a common situation when studying migrant populations. The native speaker groups (Finnish origin, Russian foreign-born and English foreign-born) filled the test in their mother tongue and include both local and migrant-origin populations. The combination of nativity of language and foreign/Finnish background allows the study of validity from a more comprehensive and practical approach.

Item response distributions were examined visually and separately for each subsample. Internal consistency of the scale was analyzed via Cronbach’s alpha, which is the lower bound estimate of reliability [35].

Testing the unidimensionality assumption is a requirement for computing sum-scores of a questionnaire [36] and for implementing unidimensional Item Response Theory models. We calculated the *u* index [37], which measures unidimensionality as the product of congeneric fit index and τ statistic. The former quantifies how the observed item correlations match the one-factor model, while the latter measures the residual between model-implied and observed correlation matrices. Unidimensional tests of 12 categorical items are expected to show *u* values exceeding 0.73 for low and mixed factor loadings, and above 0.93 for medium and large factor loadings, in samples of size *n* = 500 and larger.

In the calculation of coefficients of internal consistency and unidimensionality, the opt-out answers had to be removed to avoid biasing correlation matrices as opt-out responses are nominal in nature (i.e. their assigned value is arbitrary) and correlation coefficients rely on assumptions implying, at least, rank ordering and thus at least ordinal measurement scaling.

Before continuing with modeling, we checked whether data collection method (i.e. online and paper forms) needed to be accounted for in our models. Differential Item Functioning (DIF) has been used previously to study item-level bias for data collection method with HLS_19_-Q12 [21]. We conducted DIF analysis on our separate subsamples with the result of no item flagged for DIF, and thus we made no distinction in later analyses. For DIF analyses we used the *lordif* package for R, with Nagelkerke’s R^2^ change as criterion to compare models with a threshold of ΔR^2^ ≥ 0.020 and GPCM-based trait estimation [38].

#### Item response theory modeling

We used the Nominal Categories Model (NCM) [39] because it is a flexible IRT model with the properties of (1) naturally accommodating the multiformat items in HLS_19_-Q12, (2) allowing empirical estimation of location of all response categories (including opt-out responses) to investigate usefulness of response categories, (3) by parameterization, it might fit the data better and therefore reduce sources of item misfit found in previous validation studies [21], (4) for being a generalization of the Partial Credit Model [40, 41], which has been the choice for modeling HLS_19_-Q12 in previous studies [21, 22]. The reader can refer to *Supplementary Methods 2* for a brief technical description of the NCM.

Importantly, because the origin of parameter values of the NCM is arbitrary, a single $\:{a}_{kj}$ or $\:{d}_{kj}$ value *cannot* be considered high or low without comparison to the entire set of values for that item *j*, and parameter values shall not be compared across items [42]. Hence, item parameter values will not be directly interpreted in this study and only ordering of parameters within each item will be interpreted. For this purpose, trace lines (i.e. item probability functions) will be provided for being more informative than parameter tables showing arbitrary scaling solutions.

Response categories were reversed to align with the logic that higher choice of response categories corresponded with higher levels of health literacy. Once the model was fit to the data, we evaluated overall goodness of fit (GOF) with several indices. Note that none of the widely used and researched indices were developed ideally for the NCM, and thus our choice is based on evidence by simulation studies and suitability, considering that the scale items follow an ordinal scale in most of their distribution, while the model does not imply such restriction. The C_2_ statistic and its associated GOF indices was developed for IRT models in the context of ordinal items, high sparseness and limited-information statistics [43, 44]. The statistic C_2_ is asymptotically chi-squared under the null hypothesis that the model fits exactly in the population. In the event that the data does not perfectly fit the model [44–46], the quadratic form of C_2_ is used to calculate the root mean square error of approximation (RMSEA) to characterize the degree of model error, and thus allows approximate model fit evaluation [45, 47]. RMSEA values indicate acceptable and good fit when RMSEA < 0.08 and RMSEA < 0.05, respectively [48]. The Comparative Fit Index (CFI) is deemed good at values CFI ≥ 0.95 [49]. The SRMSR discarded in this study as it is not suited for the nominal model.

The IRT-based empirical measure of reliability was calculated [50]. Differently to Cronbach’s alpha, empirical reliability does not assume measurement error being constant for all levels of the latent trait.

Local dependence was investigated by means of the Jackknife Slope Index (JSI). Item pairs were flagged for local dependence when JSI exceeded the mean JSI value plus twice two standard deviations [51]. The JSI performs an item-level jackknife resampling procedure to compare the stability of item parameters, and by doing so emphasizes dimensionality-related violations, and thus is well suited for a multifaceted scale like the HLS_19_-Q12.

#### Item bias

In order to examine item bias we used Differential Item Functioning (DIF) and Measurement Invariance (MI) tests. Studies of MI are more heavily parametrized than DIF and thus DIF tests were implemented in case of sample size limitations.

- First, we used MI to examine equivalent functioning of the HLS_19_-Q12 splitting by native and non-native speaking of English, to test for item bias between native and acquired language skills (MoniSuomi English version, natives *n* = 379 and non-natives *n* = 1402). If evidence of measurement equivalence was found, samples of native and non-native respondents of the English HLS_19_-Q12 can be combined and compared.
- DIF analysis to examine item bias between native and migrant speakers of Finnish (Healthy Finland, *n* = 3673 and *n* = 39). Note MI was discarded due to sample restrictions.
- As a sensitivity analysis to support the previous analysis, we conducted an additional DIF test to examine item bias according to Finnish and migrant background in the Healthy Finland study: reference group was the Finnish population (answering to the Finnish version of the HLS_19_-Q12, *n* = 3673) and the focal group being the participants with a migrant background (*n* = 180).
- DIF to test response bias by reversed response scale in.
  - Finnish version of HLS19-Q12 (Healthy Finland, n=3673 and n=39, and MoniSuomi, n=2077)
  - English version of HLS19-Q12 (Healthy Finland, n=93 and MoniSuomi, n=1781)

MI was tested following the forward approach [52], by fitting nested configural (i.e. same model specification), metric (i.e. fixed slopes), and scalar full (i.e. fixed intercepts) invariance models and making nested comparisons in terms of likelihood ratio test and fit indices [53]. The model specification we tested for MI was the unidimensional NCM model previously been fit to the separate samples, and latent mean and variances were fixed to 1 throughout all models. MI is examined through models’ overall goodness of fit (indices must remain within acceptable values) and by comparing change in fit indices between nested models whose parameters are progressively constrained to equality between the tested groups. RMSEA ought to show changes (Δ) ΔRMSEA < 0.015 for consequently nested models and samples *n* > 300 [54]. The change in CFI (ΔCFI) is to remain below the cut-off value of ΔCFI< -0.01 [54].

DIF analyses were based on the framework developed by Choi and colleagues [38]. Because the framework uses a proxy to scale scoring, we used the four-point rating following standard scoring of the HLS_19_-Q12 scale (i.e. handled opt-out responses as missing). The investigation included testing for item-level uniform and non-uniform DIF. We used Nagelkerke’s *R*^*2*^ changes Δ*R*^*2*^ ≥ 0.02 as threshold for DIF flagging and trait estimation by means of the GPCM [38].

### Phase IV: scale scoring and criterion-related validity

Two standard scoring systems have been developed for the HLS_19_-Q12: type P and type D. The type P scoring is based on a sum-score of the items’ numeric values (reversed, so that a value of 1 indicates a statement feels very difficult and hence lower sum-scores correspond to lower HL), scaled to range from 0 to 100. The type D scoring is calculated as the percentage of items with valid responses endorsing “easy” or “very easy”, and therefore is based on implicit dichotomization of the original four-point graded item scale. For a score to be computed, a maximum of 20% of invalid values is allowed (i.e. maximum of two answers left black or opted out). A categorization of the type P scores has been defined with the levels “Excellent”, “Sufficient”, “Problematic”, and “Inadequate” for practical purposes. Both “Problematic” and “Inadequate” levels of HL are flagged as *limited* HL. See [21, 55] for more details on scoring and validation.

We examined the correlations between the latent estimate of HL (i.e. IRT-based) and observed estimates of HL (type P, type D, and type P levels) by means of Pearson and Spearman (HL levels) correlation coefficients. It is expected that the less granularity of the score, the weaker the associations with the IRT-based estimand and potentially with criterion variables. Yet it is important to assess the extent of information loss, since more intuitive and simplified indicators tend to become popular in literature.

Criterion related validity was investigated by comparing the IRT-based latent trait estimates (referred to as θ for convenience, although it is more precisely $\:\widehat{\theta\:}$), the type P, type D, and type P HL levels in their relationship with theoretically derived factors associated with HL. As previously found by other studies, we expect to find a social gradient of HL with self-reported socioeconomic factors. Note that the aim in criterion-related validity is checking whether the scores associate with other variables as expected to by theory, and thus associations are kept bivariate and there is no controlling or adjustment involved. A series of regression models were fit to investigate factor-specific associations, in which the dependent variable was each of the four estimates of HL, and the independent variable was one of the following at a time:

- *Socioeconomic factors*: *Sex* (register-based, male/female), *age* (register-based, coded into four groups: aged 20–34, aged 35–49, aged 50–65, aged 66–74), *educational attainment* (self-reported, harmonized between studies and coded as primary or less, secondary and higher education), *main economic occupation* (self-reported, coded as employed, student, retired, and other), *economic deprivation* (self-reported, binary coded as “yes” if endorsed at least one of the following: in the past year, feared running out of food before having money to buy more, been unable to buy medicine, or not visited a doctor because of lack of money), *foreign background* (register-based, binary as a function of both country of birth and mother tongue).
- *Health-related factors*: *self-rated health* (self-reported, dichotomized into “average or worse” versus “good or very good”), *chronic disease* (self-reported, binary and referring to diagnoses by doctor in the past 12 month period), *healthcare use in the past 12 months* (self-reported, binary yes/no), *daily smoking* (self-reported, binary), *obesity* (measured by personnel in health examination, binary cutoff BMI > 30, only available for Healthy Finland study), *Risk score for Cardiovascular Disease* (CVD, measured by personnel in health examination), only available for Healthy Finland study. This index is a sum-score from 0 to 4 including the following binary variables:
  - Dyslipidemia: if serum total cholesterol ≥ 5 mmol/l and/or LDL ≥ 3 mmol/l and/or HDL <1.2 mmol/l, and/or triglycerides > 1.7 mmol/l, and/or (register-based) use of statin medication.
  - Abnormal glucose metabolism if (self-reported) use of diabetes medication, and/or HbA1c ≥48 mmol/l, and/or glucose ≥ 7,0 mmol/l.
  - Hypertension if systolic blood pressure ≥ 140 mmHg, and/or diastolic blood pressure ≥ 90 mmHg, and/or (self-reported) blood pressure medication.
  - Obesity if BMI ≥ 30mg/m2, not computed for females after 20th week of pregnancy.
- *Migration-related factors*, only available in the MoniSuomi study: *length of residence in Finland* (register-based, categorized into 1–4 years, 5–10, 11–20 and > 20), *Finnish/Swedish language proficiency* (self-reported, categorical with values “none”, “basic”, “intermediate”, and “advanced”), *lack of a common language as an obstacle in healthcare services in the past 12 months* (self-reported, dichotomized, coded as “yes” for categories “always” or “most of the time”, and “no” when reporting “sometimes” or “never”).

The model coefficients are presented (linear estimates and odds ratios for logistic models) with their associated p-values. Population weights were used in item distribution and regression analyses. Analyses were conducted using R software version 4.4.2 [56] and the packages *dplyr* [57], *psych* [58], *likert* [59], *survey* [60], *lordif* [38], and *mirt* [50]. Results are reported with two decimals’ precision with the exception of some indices which are commonly evaluated with three decimal precision.

## Results

### Item response distributions

**Fig. 1:**
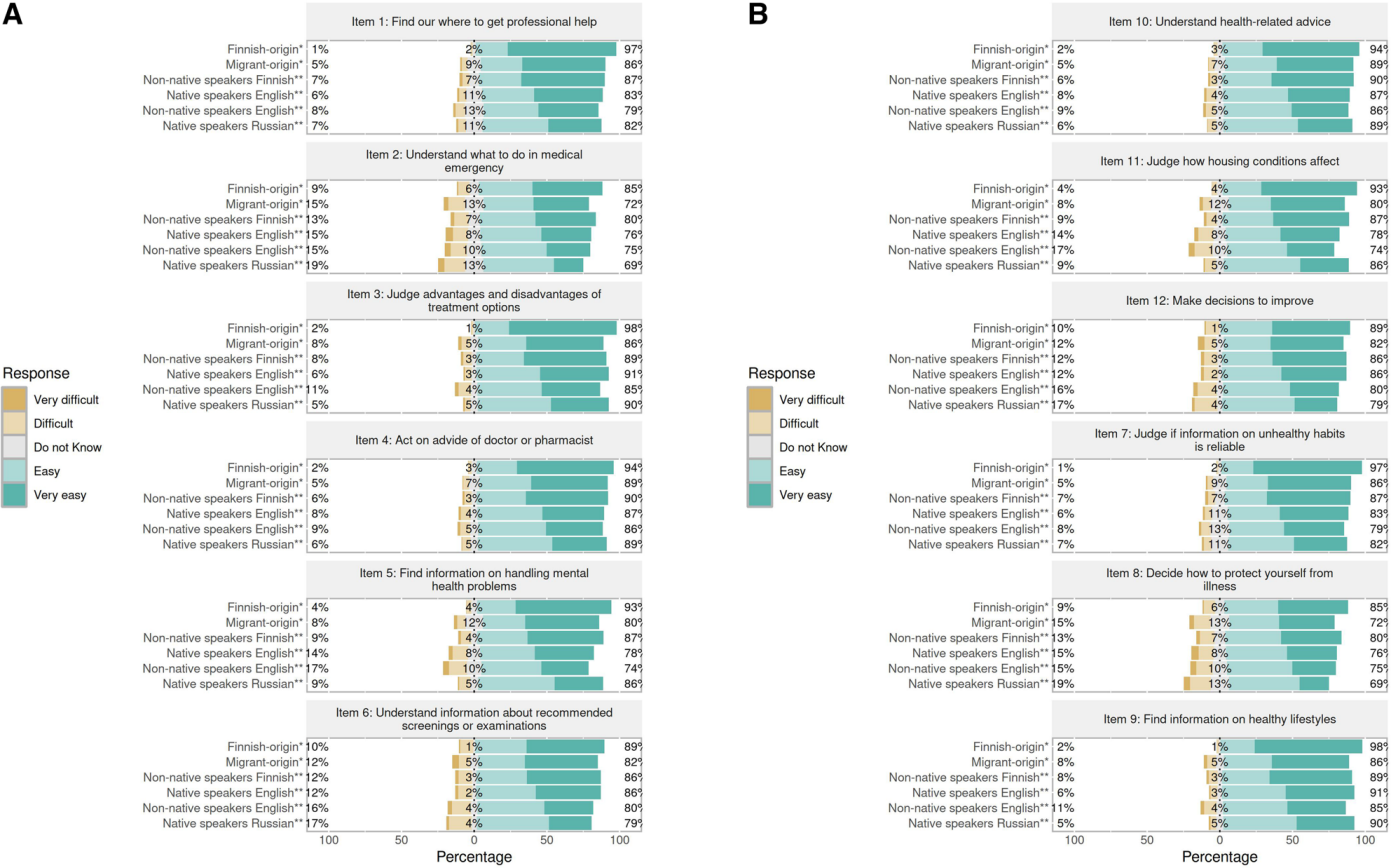
**A** Raw item distributions for HLS19-Q12 items 1 to 6, grouped by the subsamples in this study. **B** Raw item distributions for HLS19-Q12 items 7 to 12, grouped by the subsamples in this study. *Healthy Finalnd study, **MoniSuomi study

Item response distributions are showed in Fig. 1A and B, while sample sizes and further descriptives are found in Table 2. As can be seen, items had a high endorsement of upper categories of HL (i.e. “very easy” and “easy”), and low endorsement of lower categories (i.e. “very difficult” and “difficult”). This was particularly true for the Finnish general population sample, suggesting that the Finnish speaking general population may show higher scores on HL than the migrant population of Finland (i.e. all other samples in the study). The combination of the upper categories of HL (i.e. “very easy” and “easy”) ranged between 85% (items 2 and 8) and 98% (items 3 and 9), pointing at a clear ceiling effect in the Finnish-origin sample. In this sample, the two lowest categories were particularly infrequent, with combined prevalence ranging from 1% (items 1 and 7) to a maximum of 10% (items 6 and 12). Distributions showed overall more variability in migrant-origin samples, with a growth in both lower categories (e.g. native Russian speaking sample ranging between 6 and 19%) and opt-out (e.g. native Russian speaking sample ranging between 4 and 13%).

**Table 2:** Psychometric indices for HLS_19_-Q12 and the unidimensional nominal categories model fit separately to each study subsample

	Native speakers, Finnish (Healthy Finland)	Non-native speakers, Finnish (Healthy Finland)	Non-native speakers, Finnish (MoniSuomi)	Native speakers, English (MoniSuomi)	Non-native speakers, English (MoniSuomi)	Native speakers, Russian (MoniSuomi)
Classical Test Theory statistics						
*u* index	0.98	0.97	0.99	0.94	0.96	0.92
Cronbach’s Alpha	0.94	0.96	0.96	0.94	0.94	0.93
Unidimensional Nominal Categories Model						
C_2_	197 (df = 19, *p* < 0.001)	24.4 (df = 18, *p* = 0.144)	71.1 (df = 18, *p* < 0.001)	18.6 (df = 18, *p* = 0.417)	77.1 (df = 18, *p* < 0.001)	42.3 (df = 18, *p* < 0.01)
CFI	0.993	0.997	0.998	1	0.995	0.995
RMSEA [95% CI]	0.051 [0.044, 0.057	0.044 [0, 0.085]	0.038 [0.029, 0.047]	0.009 [0, 0.047]	0.048 [0.038, 0.060]	0.038 [0.023, 0.053]
IRT-based empirical reliability	0.858	0.901	0.901	0.905	0.901	0.874
Test information	15.70	26.40	37.70	27.11	23.53	23.39
Correlations between estimands of HL						
cor(θ, type P)*	0.97	0.98	0.97	0.96	0.98	0.93
cor(θ, type D)*	0.61	0.78	0.76	0.69	0.78	0.70
cor^a^ (θ, limited HL)*	-0.80	-0.87	-0.88	-0.79	-0.84	-0.69

*Note. All correlations were statically significant at p<0.01

^a^Spearman correlation coefficient

The opt-out category (i.e. grey section of the distribution bar, centered for convenience) was particularly popular among the migrant groups, and overcame the upper HL categories for the case of some items, such as items 1, 4, 5 and 7, 10 and 11 in the case of Healthy Finland migrant sample (Fig. 1A and B). Items 1, 2, 5, 7, 8 and 11 showed opt-out rates exceeding 10% in migrant samples. Opt-out was less frequent in the Finnish-origin sample, ranging from 1% (items 3, 6, 9 and 12) to a maximum of 6% (item 2 and 8). Yet opt-out was more popular than the two lowest categories combined (i.e. “very difficult” and “difficult”) in items 1, 4, 7, and 10.

The unidimensionality index (Table 2) indicated that the data conforms to unidimensionality assumptions required by the unidimensional NCM. Cronbach alpha showed very good reliability (Table 2).

### The nominal categories model: study of response categories

All models converged normally. The model corresponding to the general Finnish population required some adjustment of the parameterization for item 4, as it had zero endorsement of the category “very difficult”. For this reason, that item required one parameter less than the rest.

All models showed good to acceptable fit by means of RMSEA and CFI (Table 2). The C_2_ values seemed to be affected by sample size, since the null hypothesis of perfect fit was retained only for the small sized datasets. The JSI values flagged consistently several item pairs: the pair composed by items 3 and 5 showed local dependence for all models excepting that of Healthy Finland foreign sample. Other two pairs were items 5 and 6 (MoniSuomi Finnish non-native and MoniSuomi English native subsamples) and items 5 and 8 (Healthy Finland foreign sample and MoniSuomi English non-native samples). The pair of items 3 and 8 showed local dependence in the MoniSuomi Russian version. Additionally, the Healthy Finland migrant origin sample showed local dependence for item pairs 2 and 7, 5 and 7 and 7 and 8.

**Fig. 2:**
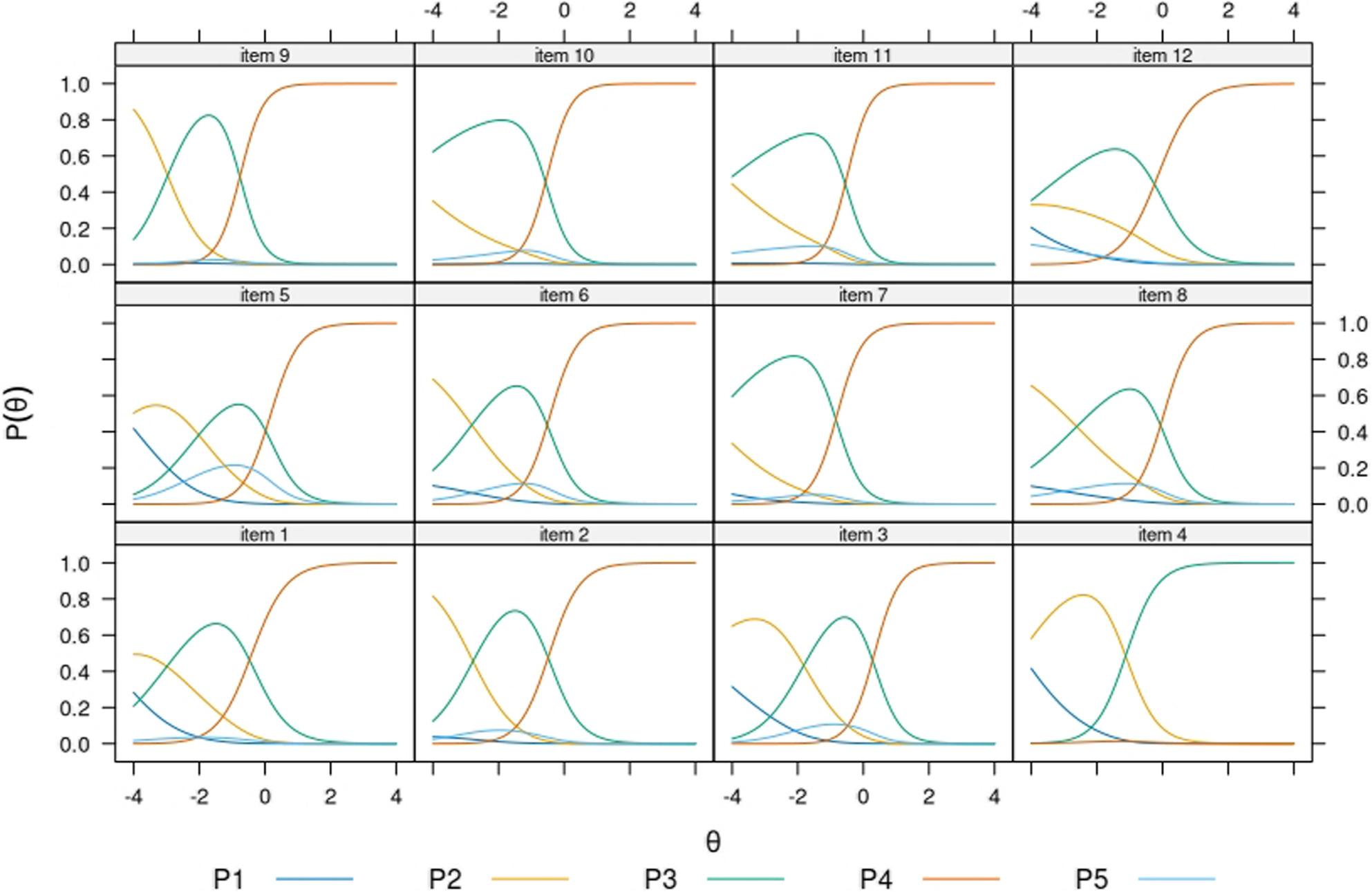
Item probability functions for the Nominal Categories Model, HLS19-Q12 items (subsample: Native speakers of Finnish from the Healthy Finland study, n= 3673) Note. Item 4 had one parameter less because the response category “very difficult” had zero frequency in this sample. As a result, the coloring of its trace lines follows a different legend. P1 to P5 acronyms in legend belong to item probability functions (P) 1 to 5

**Fig. 3:**
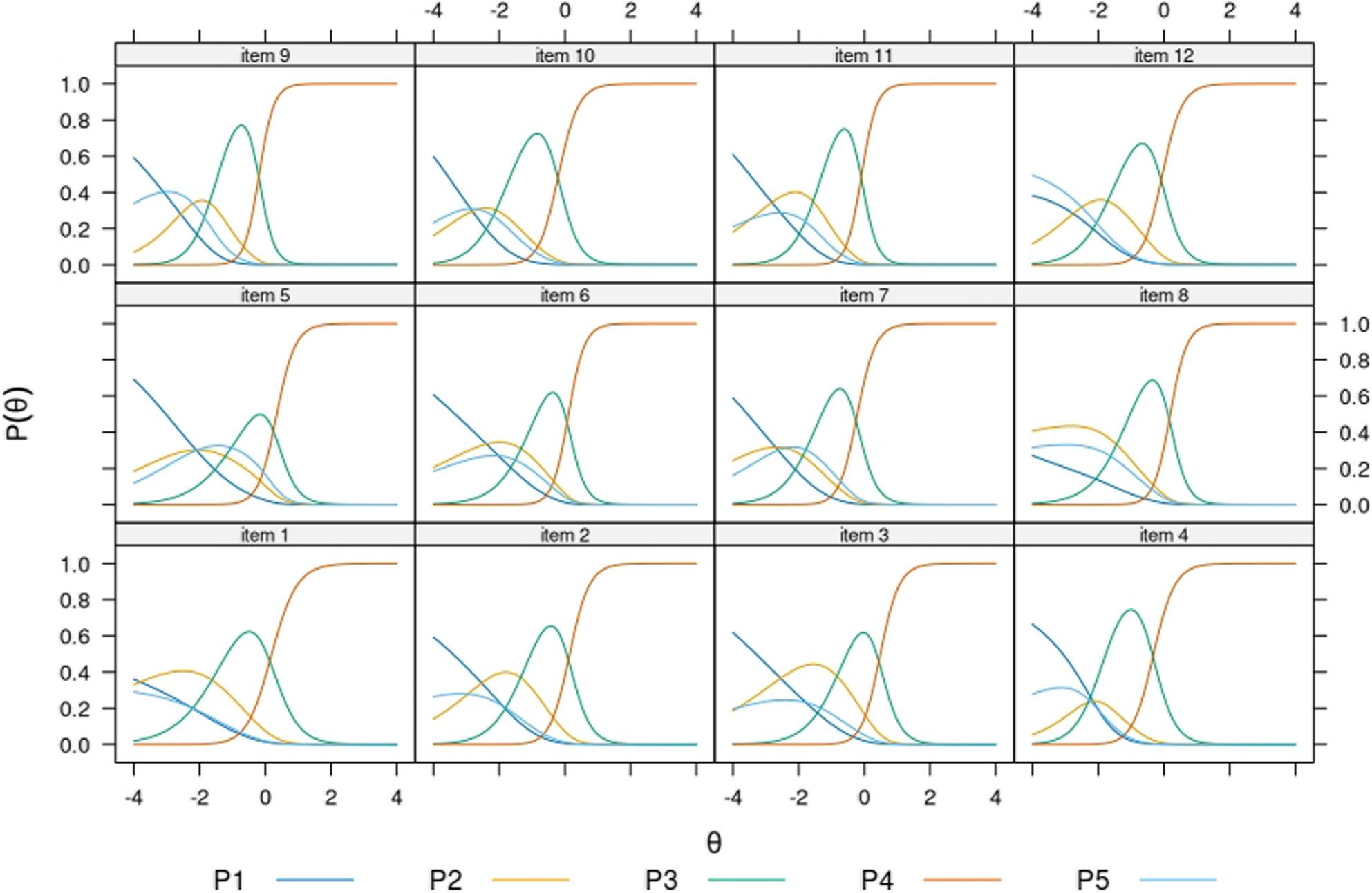
Item probability functions for the Nominal Categories Model, HLS19-Q12 items (subsample: Non-native speakers of Finnish from the MoniSuomi study, n= 2077) Note. P1 to P5 acronyms in legend belong to item probability functions (P) 1 to 5

**Fig. 4:**
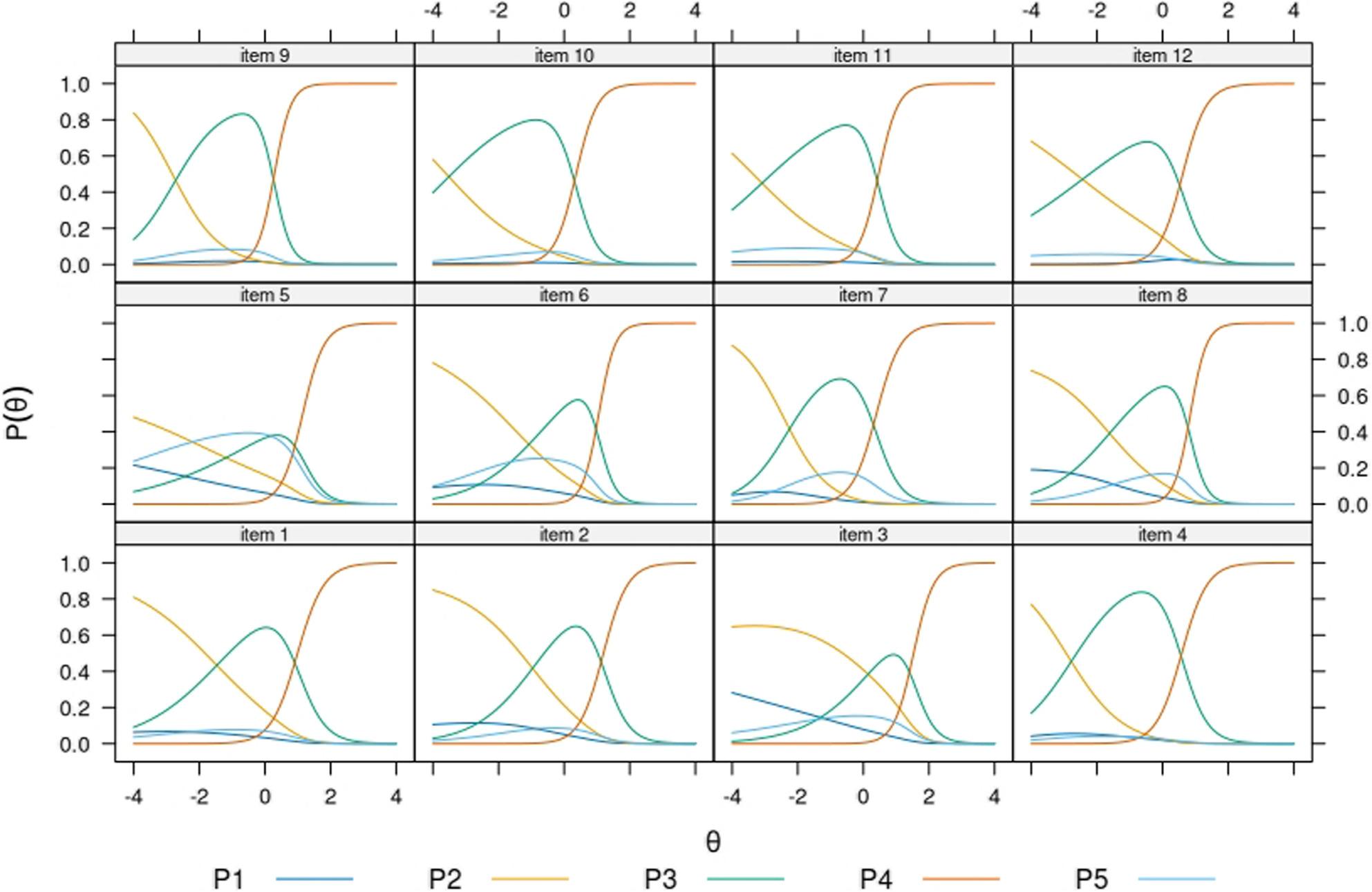
. Item probability functions for the Nominal Categories Model, HLS19-Q12 items (subsample: Native speakers of Russian from the MoniSuomi study, n= 926) Note. P1 to P5 acronyms in legend belong to item probability functions (P) 1 to 5

All items showed the expected ordering of graded categories (i.e. categories first to fourth) throughout subsamples. In most items, the highest category “very easy” showed dominance already at average levels of θ. The opt-out category showed location at different points of the continuum across the subsamples. For example, in the Finnish native model’s (Fig. 2) respondents opting-out were expected to have higher levels of the trait, as compared to the sample of migrant origin respondents who replied in the same language (Fig.3). Among respondents in Russian, the opt-out category showed the closest functioning to the Finnish native population. Item 5 showed consistently the most mixed trace lines, being the opt-out category as likely to be endorsed as either of the two low rating scales at very low levels of the trait. In most items and across subsamples, the trace lines corresponding to the categories “difficult”, “easy”, and “very easy” were most dominant across the θ interval − 4 to 4, suggesting that the “very difficult” response category is only endorsed at extremely low levels of the trait and very exceptionally. In the Finnish native sample (Fig. 1) and the Russian native samples (Fig. 4) several items function in practice as dichotomous scales starting at θ=-2.

The test information functions indicated that the scale was most informative at low and medium levels of the trait continuum. Compared to the rest of the samples, the test information was lower for the Finnish native subsample (Table 2). Most likely this is explained by a lesser overlap between person and item parameters.

### Item bias

**Fig. 5:**
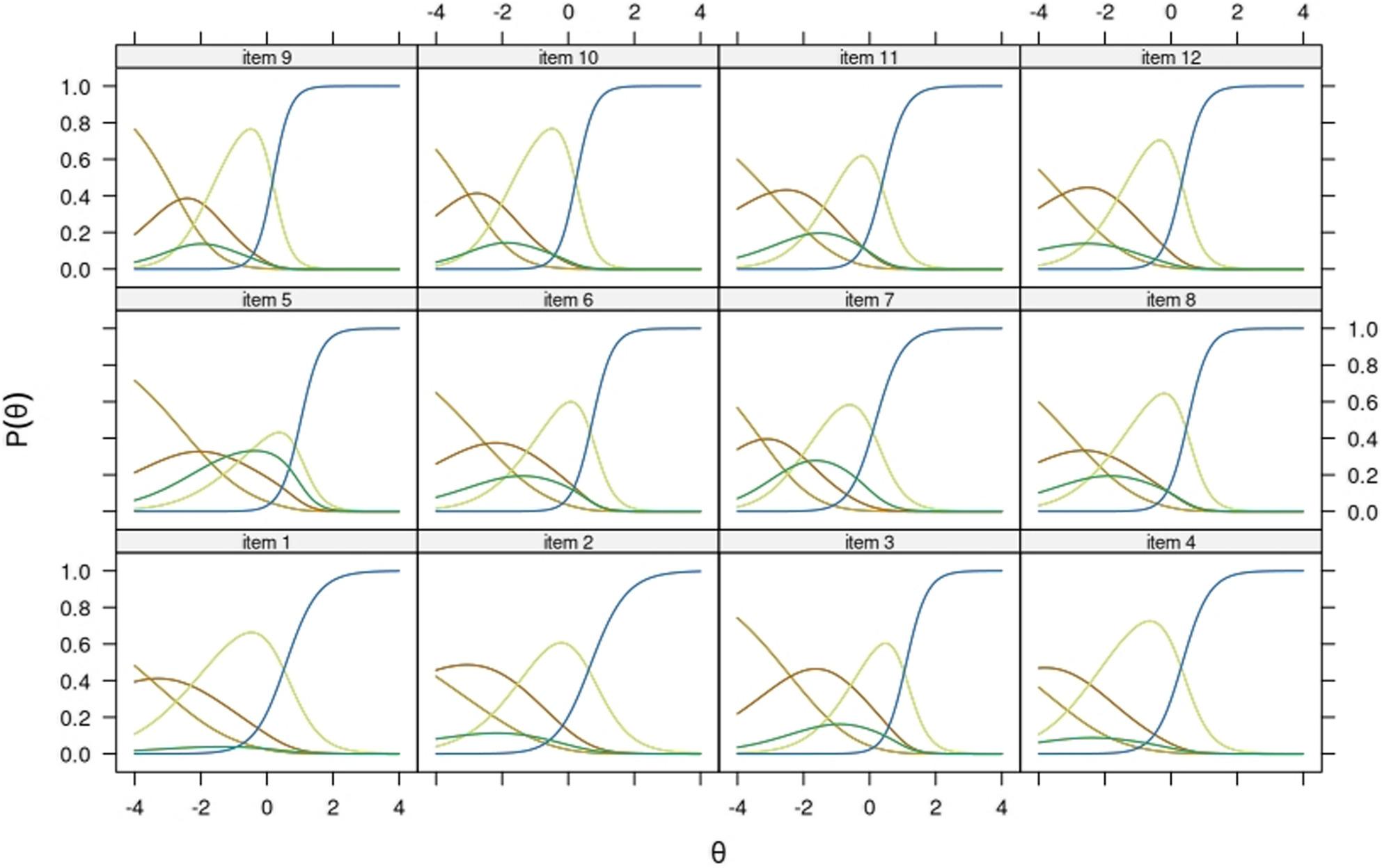
Trace lines for the Fully Measurement Invariant Nominal Categories Model, HLS19-Q12 items (subsample: Native and Non-native speakers of English from the MoniSuomi study, n= 1781)

When testing for MI of the scale among native/non-native speakers of English, the configural, metric and full invariance models showed values of RMSEA = 0.0306, 0.0311, and 0.0329, and CFI = 0.996, 0.994, 0.983. Cutoff values were not exceeded, and therefore the full invariance model was retained with identical parameterization for both groups. This result suggests that the English version of HLS_19_-Q12 can be safely used to compare scores across native and non-native speakers of English. The trace lines for inspecting visual the measurement invariant model are presented in Fig. 5. For the Finnish case, obvious sample restrictions led us to test for DIF between native/non-native Finnish speakers. No item was flagged for DIF. A sensitivity analysis was conducted in which all migrant-origin respondents of the survey were combined to increase stability through sample size. This allowed to inspect more generally potential item bias according to Finnish background (Finnish/migrant grouping). This analysis also resulted in no item flagged for DIF (i.e. all Δ*R*^*2*^ < 0.020).

To analyze item bias induced by response scale direction, we tested two language versions. The DIF analyses of the English version flagged items 3, 4, and 5 for uniform DIF (Δ*R*^*2*^ = 0.038, Δ*R*^*2*^ = 0.024, and Δ*R*^*2*^ = 0.039, respectively). In the corresponding DIF analysis conducted for the Finnish version of the scale, the item 3 was flagged also for uniform DIF (Δ*R*^*2*^ = 0.032). Results indicate that direction of the response scale does have an association with some of the observed item scores. These results argue against comparing scores obtained with reversed rating scales (i.e. comparing Healthy Finland and MoniSuomi surveys), particularly with regard to the abovementioned items.

### Scale scores and criterion validity

Table 2 shows correlations between the latent score (θ) and the HL standard scoring measures. In subsample-separated analyses of variance, all categories of HL showed the expected gradient and were significantly different from the stablished reference category “Inadequate” (all *p* < 0.001).

Criterion-related validity is examined in Tables 3 and 4. Results for the Finnish general population (Table 3) and the Finnish migrant-origin population (Table 4) showed consistent patterns corresponding to socioeconomic criteria (i.e. lower for the elder and for the economically disadvantaged, higher for females, and for those with secondary education and above) and for very good/good self-rated health. The most remarkable differences between the general Finnish and the migrant groups are the association of daily smoking with HL (association observed for Finnish population, not observed among the migrant-origin), and that of chronic conditions, which showed a reversed pattern (i.e. Finnish general population with chronic conditions present higher HL than those without, while migrants with chronic conditions showed lower HL). Use of healthcare services in the past 12-month period did not show a particularly consistent association with HL, but the two statistically significant coefficients suggest a positive direct association (i.e. limited HL showed reduced healthcare use in the general population, while migrants using healthcare showed higher θ).

**Table 3:** Model coefficients for the bivariate association between criterion variables and health literacy: Finnish general population (Healthy Finland survey, population-weighted models)

	θ^a^ (β)	*P*-score (β)	D-score (β)	Problematic or inadequate HL (OR)
Female (ref. male)	0.17***	3.10***	1.88**	0.96
Age (ref. 20–34), aged 35–49	0.10	1.67	1.14	0.98
Age (ref. 20–34), 50–65	0.07	2.18*	1.97 *	0.96*
Age (ref. 20–34), 66–74	-0.20***	-1.42	0.93	0.99
Education (ref. primary or less), secondary	0.23**	2.59	0.99	1.01
Education (ref. primary or less), higher	0.46***	5.67***	1.65	0.99
Background: migrant origin (ref. Finnish origin)	-0.03	-9.63***	-8.55***	1.15**
Economic activity (ref. employment), studies	-0.11	-3.41*	-2.67	1.06
Economic activity (ref. employment), unemployment	-0.34***	-6.95***	-4.63**	1.05
Economic activity (ref. employment), other	-0.32***	-4.68***	-3.27***	1.07***
Economic deprivation: yes (ref. no)	-0.14*	-2.83**	-4.22***	1.08**
Self-rated health: average or worse (ref. good or very good)	-0.43***	-7.01***	-6.01***	1.12***
Chronic disease: Yes (ref. no)	0.14***	1.67**	1.72**	0.96**
Daily smoking: Yes (ref. no)	-0.20**	-4.11**	-4.06**	1.07
Obesity: Yes (ref. no)	-0.07*	-0.82	-0.97	1.33*
Healthcare use 12 months: yes (ref. no)	0.05	1.47	1.60	0.95*
Health examination				
CVD risk index: 1	-0.05	-1.33	-1.40	1.03
CVD risk index: 2	-0.04	-0.12	0.41	1.01
CVD risk index:3	-0.15*	-1.60	-0.46	1.03
CVD risk index:4	-0.27*	-4.67*	-5.34*	1.09*

*β* Slope estimate in general linear models, *OR* Odds Ratio in logistic regression models

^a^ IRT-based θ values obtained by the models described in Table 2

*p<0.05

**p≤0.01

***p≤0.001

**Table 4:** Model coefficients for the bivariate association between criterion variables and health literacy: Finnish migrant-origin population (MoniSuomi survey, population-weighted models)

	θ^a^ (β)	*P*-score (β)	D-score (β)	Problematic or inadequate HL (OR)
Female (ref. male)	0.14***	3.39 ***	2.76**	0.96*
Age (ref. 20–34), aged 35–49	-0.01	-0.50	0.92	0.99
Age (ref. 20–34), aged 50–65	-0.06	-0.06	1.63	0.97
Age (ref. 20–34), aged 66–74	-0.19*	-5.11*	-2.13	1.11*
Education (ref. primary or less), secondary	0.28***	6.69***	7.10***	0.90***
Education (ref. primary or less), higher	0.31***	4.65**	6.25***	0.94*
Economic activity (ref. employment), studies	-0.01	0.24	-1.71	1.03
Economic activity (ref. employment), unemployment	-0.24***	-6.44***	-7.60***	1.15***
Economic activity (ref. employment), other	-0.23 ***	-5.97***	-6.89***	1.15***
Economic deprivation, yes (ref. no)	-0.34***	-7.98***	-10.37***	1.20***
Self-rated health, average or worse (ref. good or very good)	-0.40***	-9.58***	-9.93***	1.18***
Chronic disease, yes (ref. no)	-0.13**	-3.31***	-4.14***	1.08***
Daily smoking: Yes (ref. no)	-0.08	-0.29	-2.17	0.99
Healthcare use 12 months, yes (ref. no)	0.10*	0.62	0.63	0.99
Migration-related factors				
Language proficiency (ref. none), basic	0.05	1.00	1.22	1.03
Language proficiency (ref. none), intermediate	0.21**	6.42***	7.65***	0.89**
Language proficiency (ref. none), advanced	0.60***	18.42***	16.76***	0.75***
Length of residence (ref. 1–4 years), 5–10 years	0.05	1.44	1.18	0.99
Length of residence (ref. 1–4 years), 11–20 years	0.18***	5.74***	5.56***	0.90***
Length of residence (ref. 1–4 years), > 20 years	0.28***	9.81***	10.06***	0.84***
Language as an obstacle in healthcare, yes (ref. no)	-0.75***	-19.74***	-26.27***	1.56***

*β* Slope estimate in general linear models, *OR* Odds Ratio in logistic regression models

a IRT-based θ values obtained by the models described in Table 2

*p<0.05

**p≤0.01

***p≤0.001

Within the general population, migrants showed lower HL than those of Finnish origin across all HL estimates. Note that the sample of migrants comprises participants answering multiple language versions of the scale. The CVD risk index showed the expected gradient, and persons with three to four risk factors showed lower HL (*p* < 0.05, Table 3).

Among the migrant-origin population, HL scores also showed the expected associations with the migration-related variables examined (Table 4). Immigrants living in Finland for more than 10 years showed higher HL scores in all estimates (*p* < 0.001). Similarly, migrants reporting medium and advanced skills on either of the local languages of Finland (Finnish or Swedish) showed higher HL than those with basic or none, across all HL scores (*p* < 0.001).

In general, the latent scores showed more statistically significant associations than the observed scores. The more simplified the score, the more diluted were the association patterns found. This can be observed by the tendency shown by statistically significant coefficients to disappear as we advance from one column to the subsequent one on the right. The type P scoring retained the majority of the associations found with the latent score θ and showed the same direction across the criteria.

## Discussion

In this study, we presented the Finnish version of the HLS_19_-Q12 questionnaire and studied its psychometric properties with an emphasis on validity and population diversity considerations. These included aspects such as implications of migrant background to HL, native proficiency of scale language, and external validity study of HLS_19_-Q12 standard scoring with respect to an array of relevant factors. Additionally, we implemented IRT modeling to produce knowledge on the functioning of the opt-out and graded response scales. We used two large, nationally representative samples of the general and migrant-origin working-aged populations of Finland obtained from population studies including survey, register-based and health examination data (only in Healthy Finland 2022 study).

In sum and according to our findings, the scale shows acceptable psychometric properties and its standard P-type scoring captures most the expected associations of HL with relevant factors. With respect to the response format investigations, we recommend following the four-point Likert format for reasons of balance of the response distributions and of robustness to acquiescence effects. We will next discuss in more detail our findings in the context of each study aim.

### Psychometric functioning of HLS_19_-Q12 (Study aim 1)

The analysis of item responses revealed strongly skewed distributions and increased use of the opt-out category among migrant groups compared to Finnish-origin respondents. Item distributions and their sum-scores reflected the same ceiling effect observed in 17 countries, as reported by Pelikan et al. (2022). The Finnish-origin population showed exceptionally low endorsement of the lower response categories, with only 1% to 10% combined endorsement of “very difficult” and “difficult” across items. Notably, the opt-out category was equally or more frequently selected than the two lower categories combined in six out of twelve items (Figs. 1 A and 1B).

Across all samples, items from the health promotion domain (i.e. items 9–12) exhibited particularly low difficulty rates, with combined endorsement of “easy” and “very easy” exceeding 80% across subsamples (Fig. 1B). This ceiling effect appears to compromise the informativeness of this domain. This was also evident in the IRT models, which showed a clear imbalance in item trace line dominance. Most items demonstrated strong dominance of the two upper categories beginning at θ > − 2. These left-skewed distributions were observable in both latent trait estimates and empirical sum-scores. Similar patterns have been reported in Rasch models, with low and closely clustered item difficulty parameters [21, 22].

Although such skewness may seem undesirable, it is partially justified when one of the primary goals of HL monitoring is to identify and improve low HL levels. In this context, item calibration that enhances discrimination at the lower end of the trait continuum is desirable. Nonetheless, our findings raise questions about the standard modeling assumptions regarding the latent distribution of HL. Most IRT and traditional psychometric models assume normality of the latent trait, and HL studies have generally followed this assumption. However, the latent scores from our models, empirical sum-scores, and item trace lines suggest that HL measurement may benefit from non-normal distribution modeling. While IRT models are relatively robust to skewness [61], reconsidering HL as a unipolar construct may yield more meaningful insights and reduce parameter bias due to model misspecification [62–64].

The Nominal Categories Model (NCM) provided an empirical assessment of the rating scale and yielded the expected ordering of response categories. However, the lower categories (“very difficult” and “difficult”) were located extremely low on the latent continuum and exhibited overlapping trace lines. Pelikan et al. [21] discussed collapsing response categories in pairs for similar reasons and suggested that cultural variation in response styles may justify a dichotomized format (i.e. “difficult” vs. “easy”), which aligns with the computation of the type D score. Moreover, prior research shows that the NCM can produce unordered item parameters when category endorsement is low and characterized by J-shaped item distributions [65, 66]. Pelikan et al. [21] also applied the NCM to assess category ordering and concluded that unordered categories in several countries likely stem from such distributions. In our study, setting parameter constraints successfully addressed this estimation issue, and no information was lost due to dichotomization [67]. Based on our findings, we recommend against collapsing the low response categories, as they are most useful in accurately estimating problematic levels of HL, which is a primary goal for HL measurement.

To further improve the instrument, we suggest that item trace lines should reflect the conceptual hierarchy of HL competencies, which follow a difficulty gradient (i.e. access < understand < evaluate < apply). For example, items assessing evaluation of health information should ideally measure higher levels of HL than those assessing access. In our study, this gradient was partially observable in trace lines across subsamples. However, we refrain from interpreting item parameters directly, as a known limitation of the NCM is that item parameters lack meaningful magnitude and are not comparable across items [42].

Measurement invariance analyses indicated that scale interpretations can be made consistently for both native and non-native English speakers. This property enhances the instrument’s utility for international applications and for monitoring HL in both migrant and local-origin populations. Importantly, respondents who completed the English/Finnish version chose to answer in such language voluntarily, suggesting sufficient language proficiency and confidence with the language. A similar pattern was observed for the Finnish version, although limited sample size constrained further invariance testing. These findings align with prior research demonstrating equivalent functioning of the HLS_19_-Q12 in migrant groups [68, 69] and across countries [70].

We recommend making the questionnaire available in multiple languages to minimize measurement error stemming from language proficiency differences. The HLS_19_-Q12 is already available in a wide range of languages [20].

### Opt-out response category in HLS_19_-Q12 (Study aim 2)

In large-scale population surveys, explicitly offering an opt-out response option is a measure to reduce cognitive burden of decision making [71]. In this study, however, we observed that the opt-out category was, at times, more frequently selected than the lower-end response categories (“difficult” and “very difficult”), particularly among migrant respondents. We hypothesize this reflects familiarity with the Finnish society and system in a broader sense, which is interpreted by respondents as beyond the scope of the scale and therefore is preferably opted out than considered itself as “difficult”. We support this view with several findings: (1) foreign-born respondents who chose to respond in Finnish had notably less opt-out rates and higher endorsement of upper categories (Figs. 1 A and 1B), and (2) migration-related factors in regression analyses in Table 4 are all significantly associated with HL in the direction expected by this hypothesis of familiarity with the system. In terms of the variation observed across languages in the use of opt-out, we consider this issue should be further investigated with a mixed-methods approach, if deemed necessary. Whatever the reason is for the dominance of opt-out in response distributions, it raises concerns about its impact on data quality and score interpretability and its removal seem more justified than maintaining it.

Item 5 showed overall the highest incidence of opt-outs, but elevated rates were also observed across all items in the Disease Prevention domain (i.e. items 5–8). Item 5 is the only item referencing mental health, which may partially explain its inconsistent findings. The IRT model results further suggested that the functioning of the opt-out category differed across groups in its location along the latent trait and supported the evidence on item 5 showing mixed trace lines consistently. The model results deepen further the information by raw response distributions, in suggesting that the scale functioned in practice as a three-point Likert scale for groups responding in their native language (i.e. Figures 2 and 4), and as a four-point one for more heterogeneous groups of respondents (i.e. Figures 3 and 5).

In previous sections, we argued against collapsing the two lower response categories despite their inconsistent dominance patterns. One strategy to improve their stability is to avoid the explicit inclusion of opt-out options, which appear to compete directly with these categories. Our findings align with prior research showing that the presence of a “do not know” option increases opt-out rates [72], and that providing alternative response options introduces inconsistency in the ordering of trace lines [73].

While the opt-out category may have enriched IRT-based trait estimation—particularly at the lower/intermediate end of the HL continuum—it also introduced challenges for measurement equivalence and constrained modeling choices. Its inclusion complicates assumptions of ordinal scaling and may obscure meaningful variation in low HL levels. Further research on this issue could explore the effects of including a middle category over the response distributions to replace the opt-out option. Additionally, large-scale survey studies could benefit from simulation studies on the effects of imputing over the opt-out responses, given that their actual distribution is empirically known.

### Response styles in HLS_19_-Q12 (Study aim 3)

Our findings suggest that HLS_19_-Q12 is rather robust to method factors, as more than three fourths of the items remained unflagged for DIF according to reversed ordering —3 out of 12 items in the English version and 1 out of 12 in the Finnish version showed DIF. No systematic effects were observed when comparing data collection modes (paper vs. online), indicating consistency across administration formats.

Interestingly, subsamples exposed to the reversed response scale (Healthy Finland study, starting with “Very difficult”) tended to show on average a lower prevalence of the “Very difficult” and “Difficult” response categories. This pattern contradicts the acquiescence hypothesis. These results support the continued use of positively worded items and ascending response scales in the measure. For researchers aiming to minimize acquiescence bias, IRT latent estimates are recommended [74].

Analysis of the item trace lines revealed that respondents answering in their native language—specifically the Finnish general population and Russian-speaking migrants (Figs. 2 and 4)—exhibited more similar response behavior than non-native speakers (Figs. 3 and 5). The latter groups demonstrated a more varied and balanced use of response categories across the latent trait continuum. Further attention should be paid to this pattern in future research, as it might have practical implications on whether to dichotomize the current Likert scale in HLS_19_-Q12.

### Alignment of HL scores with theoretically founded factors (Study aim 4)

In this study, we applied psychometric theory with a pragmatic focus. We obtained an IRT model-based estimand of HL under given assumptions, and then linked it to several empirically obtained estimands (i.e. type P and D scores and dichotomous “limited HL”) in line with recent recommendations [35, 75–78]. All the estimands under examination showed evidence of concurrent criterion-related validity consistent with theoretical expectations and earlier findings on HLS_19_Q12. Our results indicate that the type P score approximates the IRT-derived estimand and preserved most of the associations found, particularly in the case of health- and migration-related validators.

Since the IRT-based estimand is built on the full response scale of the items, it is expected that the P type score correlates better with the IRT-based person location estimates than the D type score, which discards information by building on dichotomized items scales. However, it is important to investigate in which ways the use of this simplified scoring system implies loosing relevant information, particularly because the dichotomized scale has been suggested as more robust to cultural effects on responding [21]. Several differences were observed across estimands and between general and migrant populations. The latent estimand showed no variation according to foreign background in the Healthy Finland study, while the sum-score based estimands did (Table 3). Although the DIF analyses did not flag any item for bias, this pattern could reflect non-equivalent functioning. Further research is necessary to confirm full equivalence of the Finnish version of the scale across population subgroups.

Associations between HL and healthcare utilization have yielded mixed results in the literature, and our findings reflect this complexity. Only problematic HL was linked to healthcare use in the previous 12-month period among the Finnish general population, which could reflect lower health status of persons co-occurring with limited HL. However, the numeric HL scores did not vary by healthcare use, consistent with prior studies [79, 80]. HL was positively associated with healthcare use among migrants, In line with previous findings [81].

Self-reported chronic conditions showed a reversed pattern of association between general and migrant populations. Migrants reporting chronic conditions showed overall lower HL and significantly higher odds of problematic or inadequate HL. Conversely, individuals from the Finnish general population with chronic conditions tended to report higher HL and were less likely to fall into problematic HL categories. In Finland, it has been previously found that foreign background can mediate the association between chronic conditions and discrimination experiences in healthcare services in the same manner [82]. It is important to note that all associations in this study were kept bivariate in line with common practice in external validation studies; thus, observed patterns may be confounded by other background factors, such as socioeconomic or demographic variables. Mechanisms explaining the associations reported (e.g. confounding or mediation) are beyond the scope of validation efforts and belong to further research addressing them.

A key contribution of this study was the examination of concurrent validity between HLS_19_ -Q12 and medical indicators. The CVD risk index showed the expected gradient in its associations with HL among the Finnish general population. Persons with highest score on the CVD risk index showed significantly higher risk of presenting problematic or inadequate HL. These findings are in line with recent studies using different measuring methodologies, which highlight the potential of HL education to improve population health outcomes [83–85]. The findings in our study are in line with the previous literature on correlates of limited HL, such as poor self-perceived health, chronic illness, living alone, and undocumented or transitional legal status.

The examination of migration-related factors revealed that higher language skills and longer time living in Finland were linked to more favorable HL estimands, in line with earlier studies [17, 18, 86–88]. Both variables emphasize the relational scope of HL and highlight the importance of gaining familiarity with the healthcare system of the new country and accessing health-related information for better HL outcomes, which are well-known factors within migrant populations [89]. A study examining HL and health disparities among migrants in Europe found that the higher the country’s HL, the smaller the health gap between the local-origin and migrant populations [90]. The study discussed the role of integration policies in shaping health disparities and HL in the countries examined. Our study lends further support to the adoption of culturally and linguistically responsive policymaking, which has the potential to enhance health literacy among migrant populations and, in turn, advance health equity for all. Such approaches are consistent with the Health in All Policies framework and represent a necessary step toward building more inclusive and resilient public health systems.

### Study limitations

Some of the challenges of this study pertain to the measurement of HL in migrant populations, such as measurement error due to item interpretation. For instance, answering to HL items referring to the country where the person lives, as compared to referring to their original country, or to a more broad interpretation where all healthcare information and services accessible are taken into consideration [91]. Moreover, language complexities may increase the rate of opting out among migrant respondents, particularly if that is reflected as a valid option to choose from.

This study was conducted within the Finnish healthcare and sociocultural context, which may limit the generalizability of findings to other countries. Finland’s relatively high baseline HL, universal healthcare coverage, and migrant integration policies shape both access to services and HL outcomes. As such, the psychometric performance of the HLS_19_-Q19 and its associations with health-related variables may differ in settings with distinct health systems, cultural norms, or levels of institutional support for migrants. Future research should replicate these analyses in diverse international contexts to assess cross-national applicability and ensure culturally responsive HL measurement.

Sample sizes of the study groups limited further use of the measurement invariance testing and were thus addressed via DIF. For instance, it was not possible to include in our analyses the Swedish version of the scale, despite of Swedish being an official language of Finland (*n* = 175). This limits generalizability across all migrant-origin populations. Our results call for more conclusive invariance studies e.g. according to Finnish background (Finnish/migrant grouping), despite of DIF analysis shedding adequate results. Finally, DIF analyses revealed few items showed bias across study groups, which might bias sum-scores to a varying extent.

## Conclusions

This study made several important contributions to the measurement of HL. A Finnish-language version of the HLS_19_-Q12 was developed and its psychometric properties were found to be acceptable. Specific recommendations were made regarding topics relevant to measuring HL internationally, such as response formatting, item bias, study of cultural effects over response style, use of scale scores, and invariance issues affecting scale interpretation. Our findings and reflections are applicable more broadly to practices on public health surveillance. In terms of HL, this study found evidence of external validity based on associations with socioeconomic, health- and migration-related factors. Our findings underscore the development of culturally and linguistically sensitive intervention programs to increase HL among different groups of migrants. Areas for refinement and future investigation were also identified. To monitor and promote effectively the HL of the general and migrant populations, it is fundamental to strive for valid measurement tools and to adopt culturally and linguistically conscious policymaking.

## Supplementary Information

Supplementary Material 1

## Data Availability

The data in this study may be available upon request, issued and granted by the Finnish Institute for Health and Welfare.

## Abbreviations

cor: Correlation
CVD: Cardiovascular Disease
df: Degrees of freedom
DIF: Differential Item Functioning
GPCM: Generalized Partial Credit Model
HL: Health Literacy
HLS_19_-Q12: Health Literacy Survey Questionnaire, 12-item version
IRT: Item Response Theory
MI: Measurement Invariance
NCM: Nominal Categories Model
OR: Odds Ratio
