# Supplementary material for: Health equity through health literacy: validating HLS19-Q12 in general and migrant-origin populations of Finland
Source: BMC Public Health. 2026 Jan 27;26:658. doi: 10.1186/s12889-026-26356-x (PMC12918337; doi:10.1186/s12889-026-26356-x)
Supplement: Supplementary file 1 — Supplementary Material 1 [file 12889_2026_26356_MOESM1_ESM.docx]

**Supplementary Methods 1: Methodological considerations in the Study**

Functioning of the “opt-out” response option

In the HLS_19_-Q12, it is possible to include an opt-out response option (i.e. “I do not know”) allowing respondents to skip rating the ease of a statement. Research has discussed the benefits of including an opt-out response to reduce the cognitive burden of respondents’ decision making [30]. In HLS_19_-Q12, it is handled as a missing value, and the respondent’s score is calculated if the total amount of blank and opt-out responses is 20% or less (a maximum of two items in the 12-item questionnaire). The opt-out response category has not previously been analyzed in HLS_19_-Q12 studies, likely due to its infrequent use or absence from the scale. In the data collected in Finland, however, the opt-out option was frequently selected (e.g. 13% of respondents answering in Finnish chose to opt out of item 5, while 25% of migrants did so when answering in English). Moreover, opting out showed higher frequency than the “very difficult” response option across multiple items, where “very difficult” responses remained below 10%. In the samples of this study, an overwhelming majority of scores that were not calculated by the 20% cutoff were caused by choosing to opt-out rather than item non-response. Certainly, we cannot truly estimate the extent of increase in non-response if the opt-out option was removed. In any case, a response category with such an impact on scoring warrants specific investigation.

Item format and implications on modelling

The opt-out category poses a challenge to existing approaches for the HLS_19_-Q12, because this response category does not follow any gradient (i.e. cannot be handled as part of the same ordinal scale as the other four graded options). Moreover, its placement at the end of the scale eliminates any centrality effects.

Validation studies of the HLS_19_-Q12 have consistently supported its psychometric properties in multiple countries [22, 31–36]. However, previously used models in such studies cannot fully accommodate the response format of items with opt-out category (i.e. worded as “I do not know”) combined with the four-point rating scale. To date, its impact on the overall scale and psychometric properties remain unexplored. In this study, we investigated the HLS_19_-Q12 by implementing the Nominal Categories Model (NCM)[37, 38] to better understand how the scale functions across different samples and language versions. The NCM is indicated for multiple aspects that this study directly addresses, such as “for (a) testing the assumption of ordered response categories inherent in most psychological scales, (b) testing the assumptions (…) inherent in more restricted models (…), (c) identifying poorly functioning response categories that should be collapsed, (d) conducting a fine-grained study of cross-cultural differences in category usage, and (e) studying the effects of reverse wording” [39].

Cultural differences and response style: acquiescence.

The HLS_19_-Q12 was introduced by the following statement: “How easy would you say it is to…?”, followed by a four-point rating scale starting with “Very easy” and finishing with “Very difficult” and a final “Do not know” opt-out category. Cultural differences in response behavior may introduce systematic variation unrelated to actual HL levels. One possible way in which response styles could affect observed item distributions is acquiescence—the tendency to agree regardless of item content. Differences in response style are one of the main sources of bias in cross-cultural measurement [40], and its impact has not been examined for HLS_19_-Q12.

The datasets of Healthy Finland and MoniSuomi studies are otherwise highly comparable but included response categories of the HLS_19_-Q12 with reverse ordering. This allows comparison of response distributions between language versions in Finnish and English, and between migrant and general populations. All formats had the opt-out category (“I do not know”) as their fifth category, after the four Likert-type response categories and at the end of the rating scale. We investigated the effect of response style by comparing response distributions and testing for IRT-based measurement invariance.

Use of scale scores

The HLS_19_-Q12 is required to be both psychometrically sound and convenient to use for monitoring HL in practice. Validating the sum-score is fundamental for a tool to become widespread in the applied context, where sophisticated modeling of scale scores, such as routine IRT-based estimation of HL as a latent trait, is unrealistic. We agree that “one should engage in psychometric work of various kinds to ensure that sum scoring is a reasonable approach for a particular set of items” [41]. Despite its limitations, the observed sum-score approximates the true sum-score. This study examined the association between different methods for HL scoring and factors expected to vary according to HL. Associations in the theoretically expected direction would imply evidence of concurrent criterion-related validity (e.g. low scores in HL correlating with poor scores in health-related criteria or low socioeconomic status). We compared criterion-related validity of standard scoring of HL, which are based on sum-scores, and the IRT-based estimand of HL. An array of criterion variables was chosen belonging to sociodemographic factors, variables related to health and health-related behaviors, and migration-related factors.

**Supplementary Methods 2: the Nominal Categories Model**

The probability that a subject with latent trait θ (HL, in this study) will respond to item *j* in category *k* according to the NCM is modelled as follows:

$$P_{j}(k|\theta)=\frac{e^{a_{kj}\theta+d_{kj}}}{\sum_{i=1}^{m} e^{a_{kj}\theta+d_{kj}}}$$

where the summation in the denominator corresponds to all K response categories for a given item *j*. By convention, parameters refer to k=K-1 response categories, and thus parameters $a_{0j}$ and $d_{0j}$ correspond to k=1. Each response category has its own $a_{kj}$ and $d_{kj}$ parameters, which do not have the same interpretation as in the dichotomously scored models (Thissen & Cai, 2016). For a given value of θ, the response category most likely chosen will be the one with the highest numerator, because of the increasing exponential function *y=exp(x)*, and thus choice depends on values of $a_{kj}\theta+d_{kj}$. The intercept parameter $d_{kj}$ quantifies how appealing a response category independently of the value of θ is. When θ=0, the highest the value of $d_{kj}$, the more likely the response category is to be chosen. The slope parameter $a_{kj}$ indicates how values of θ relate to the probability of choosing a given response category *k.*

When scoring function values are left freely estimated, the nominal model shows the empirical location and functioning of every response function, which is particularly useful for test construction and development. However, the set of slope and intercept parameters must include anchoring conditions to be able to uniquely determine the parameter values. Additionally, the NCM can become numerically unstable if poor choices for the high and low values of parameters are chosen, resulting in $a_{kj}$ values with very large absolute values. It is recommended to choose high and low anchors that constrain estimated $a_{kj}$ parameters to fall between 0 and K−1 either by theoretical means or by re-estimating the model with better values following convergence. The response function corresponding to the highest (lowest) category monotonically increase (decrease), and reaches the asymptotic value of 1 (zero) for increasingly high (low) values of θ. All response categories in between them show unimodal distribution and, if showing equal values of $a_{kj}$they reach their maximum at the corresponding θ, regardless of their associated $d_{kj}$ values.

If the scoring function values are equal to the integer category values 0, …, K − 1, the nominal model becomes a model for graded item response data (Thissen & Cai, 2016). Consequently, we expect the models in our study to shed $a_{k}$ parameters compatible with a 4-point graded response process (i.e. *a_k0_* to *a_k3_* showing increasingly higher values) and to show trace lines where categories are arranged accordingly. In agreement with anchoring and identification arguments above, we fixed few parameters to work as scaling anchors (i.e. *a_k0_* =0, *a_K-1_*=K-1, *d_0_*=0*)*. Note that the last response category (K-1) corresponds to the opt-out option, and thus we left it freely estimated and set the value of the highest graded response slope parameter to *a_k3_*=4 instead.
